# Irrigation Challenges, Water Access, and Adaptation Strategies in Smallholder Farming: Evidence From Gaza Province, Mozambique

**DOI:** 10.1002/gch2.202500342

**Published:** 2025-11-23

**Authors:** Hercidio Jaime Tandane, Bento Filipe Francisco, Alfredo José Maholele, Manuel Mulhuli Sitoe

**Affiliations:** ^1^ Agriculture Research Institute of Mozambique Chókwè City Gaza Mozambique

**Keywords:** Gaza province, irrigation challenges, Mozambique, smallholder farming, water access

## Abstract

Irrigation is a critical component of smallholder agriculture, particularly in regions prone to climatic variability and water scarcity, such as Mozambique. This study investigated the challenges faced by smallholder farmers in accessing water for irrigation, explored the adaptation strategies they have adopted, and evaluated the effectiveness of these approaches in enhancing agricultural productivity and resilience. Using quantitative methods, the study highlights significant disparities in water access across the districts of Chókwè, Mandlakazi, and Guijá. Key findings include an ageing farmer population, limited access to efficient irrigation technologies, inadequate irrigation infrastructure, and the increasing impact of climate change, particularly in the form of droughts. Farmers primarily rely on gravity flow irrigation systems, but the lack of sufficient water storage and drainage infrastructure impedes effective water use. The study also identified crucial adaptation strategies, including the rehabilitation of irrigation systems, the adoption of solar‐powered irrigation technologies, and the promotion of climate‐resilient agriculture practices. The study emphasized the need for policy interventions focused on investing in irrigation infrastructures and providing capacity‐building programs to enhance water management and climate adaptation. These findings are vital for informing policies aimed at improving water access and resilience in smallholder farming systems in Gaza province and similar regions.

## Graphical abstract

This study examines irrigation challenges faced by smallholder in Mozambique and analyzes water access disparities across the Chokwe, Mandlakazi, and Guija districts. Findings highlight ageing farmers, limited irrigation technologies, poor infrastructure, and climate‐change‐driven droughts. Effective adaptation strategies, including rehabilitated systems, solar irrigation, and climate‐resilient practices, underscore the need for policy investment in irrigation infrastructure and farmer capacity building.

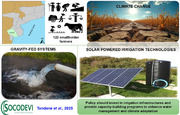

## 1 Introduction

Water access for irrigation is a critical determinant of smallholder farming productivity, particularly in regions such as the Gaza province and Mozambique, which are vulnerable to climate variability and water scarcity. Irrigation is vital for stabilizing crop production and enhancing food security in these areas, yet many smallholder farmers face persistent challenges in securing reliable and sufficient water sources. In Gaza province, limited infrastructure, financial constraints, and governance challenges hinder the development of sustainable irrigation systems, leaving farmers struggling with water shortages and unreliable water sources [1, 2]. The reliance on rainfed agriculture further exacerbates the vulnerability of smallholder farms to drought and changing weather patterns [3].

The role of irrigation in improving agricultural productivity and mitigating the impacts of climate change has been widely documented [4, 5]. Similarly, a growing body of research highlights the adaptation strategies that smallholder farmers employ to sustain their livelihoods in the face of increasing climate‐related risks [6]. However, a critical gap remains in understanding how smallholder farmers in Gaza province specifically navigate these water access challenges and adapt their practices. Existing studies tend to focus on infrastructural and financial barriers but provide limited insight into how local communities engage with governance mechanisms, institutional support, and farmer associations to develop context‐specific adaptation strategies [7, 8, 9].

In this study, adaptation strategies are defined as the adjustments in farming practices, resource management, and institutional engagement that farmers adopt in response to irrigation constraints and water scarcity. By focusing on the interplay between water access challenges and adaptation practices in Gaza province, this research provides novel empirical evidence from a context that has received limited attention in the adaptation literature. Using quantitative approaches, the research examined infrastructural deficiencies and community‐driven strategies aimed at enhancing irrigation accessibility. In doing so, it contributes to ongoing debates on smallholder resilience by highlighting the specific dynamics of irrigation governance and farmer‐led adaptation in water scarce region of Mozambique. The findings aim to inform policymakers, development practitioners, and stakeholders on sustainable water management strategies tailored to the needs of smallholder farmers in water‐scarce regions [10, 11].

## 2 Study Area and Context

This study was developed in the districts of Chókwè, Guijá, and Mandlakazi, all located within the Limpopo River basin in southern Mozambique. The basin plays a pivotal role in regional agriculture, livelihoods, and water management. These districts are predominantly agro‐pastoral and characterised by a semi‐arid to sub‐humid climate, with low and irregular rainfall, high temperatures, and frequent droughts. These climate features, coupled with socio‐economic vulnerabilities, make the region particularly sensitive to climate change and water‐related stresses.

The Limpopo River and its tributaries are the principal surface water bodies in the region, supplying essential water for irrigation, domestic use, and livestock. In addition to surface waters, the districts also rely on shallow aquifers and hand‐dug wells for local irrigation and domestic water use. Deep groundwater exploitation remains limited due to infrastructure and financial constraints [12]. The region has a long‐standing history of irrigation development, particularly in Chókwè, where formal irrigation schemes date back to the colonial period. Governance of irrigation systems varies across districts, involving a mix of government‐led, community‐managed, and donor‐supported schemes. These arrangements affect access, efficiency, and sustainability of irrigation practices [13].

### 2.1 The Chókwè District

The district is in the southern part of the Gaza province. The climate is semi‐arid, characterized by an annual average temperature of 22°C to 26°C and annual rainfall of about 500 to 800 mm. The soil is predominantly alluvial and red clay [14]. The district benefits from the lower Limpopo Irrigation Scheme, one of the country's most extensive formal irrigation networks, supporting both commercial and subsistence farming [15]. The population is approximately 222 396 inhabitants, including 122 755 women and 99 755 men, with 14 490 small to medium subsistence farmers cultivating an average of 2.1 hectares (ha) of land and benefiting from government extension services [16]. The main crops grown include cassava, sweet potato, cowpea, maize, and rice. Livestock such as sheep, cattle, goats, and poultry are also essential sources of livelihoods [17, 18].

### 2.2 The Guijá District

It is in the southern part of the Gaza province. The district has a semi‐arid climate, with an annual average temperature ranging from 24°C to 26°C. Annual rainfall ranges from 400 to 500 mm, and the predominant soil types include alluvial, sandy, red, and firm clay soils [19, 20]. Water access for agriculture is limited to shallow wells and seasonal streams. No formal irrigation system exists, but small‐scale farmers rely on informal irrigation practices supported by NGOs or private actors [21].

In 2017, the total population was approximately 104 540, with 44.65% men and 56.4% women. Among them, 6040 were small producers cultivating an average of 2.9 ha of land, supported by private or government extension technicians. The major crops grown include sweet potatoes, maize, beans, and cassava. Likewise, livestock farming, including cattle, ducks, goats, chickens, pigs, and sheep, plays a crucial role in household livelihoods [14, 22].

### 2.3 The Mandlakazi District

Mandlakazi is situated in the southern Gaza province, bordered by the Panda, Zavala, and Inharrime districts, as well as the Indian Ocean. The climate varies from a dry steppe in the interior to a humid tropical region along the coast, with average temperatures ranging from 20°C to 28°C and an annual mean above 24°C [23]. The district receives around 500 mm of rainfall per year, and its soils are predominantly sandy with moderate agricultural potential [24, 25]. Water access is mostly derived from shallow groundwater and seasonal rivers, with limited formal irrigation infrastructure [26]. In 2017, the population was 140.588, with 45.8% men and 54.2% women. The economy relies on agriculture, livestock, fishing, and labor migration, with key crops including maize, cassava, beans, and sweet potatoes. Livestock farming (cattle, goats, pigs, chickens, and ducks) is essential, alongside the exploitation of forest resources for artisanal crafts, charcoal, and firewood production [27].

## 3 Research Methods

### 3.1 Selection of Farmers' Associations

The study targeted smallholder irrigation farmers in Gaza province, Mozambique, focusing on Chókwè, Guijá, and Mandlakazi districts. These districts were selected based on their reliance on irrigation farming and ongoing challenges related to water access. Farmers' associations were identified with the support of SOCODEVI, an international organisation working on the Mozambique Rural Women's Economic Empowerment project. The following farmers' associations were selected to ensure a diverse representation of farming practices and water access challenges. The selected farmer's associations included Guinguiricane, Armalugue, and Josina Machel in the Chókwè district; Nkateco and Makateco in the Guijá district; and Esperança da Vida, Árvore da Vida, Lhuvukane, and Filipe Nyusi in the Mandlakazi district.

### 3.2 Sampling Procedure

A multi‐stage sampling procedure was employed in the study. The first stage involved selecting three districts: Chókwè, Guijá, and Mandlakazi. These districts were chosen due to their irrigation‐dependent farming systems. In the second stage, farmers' associations that were actively involved in smallholder irrigation were selected, with assistance from SOCODEVI. The third stage involved selecting farmers within these associations, utilizing stratified random sampling methods to ensure diverse representation in terms of gender and farm sizes. A total of 122 farmers participated in the survey, which was determined using Slovin's formula at a 95% confidence level, following the methodology suggested by Rono [28]. At least 60% of the respondents were women, in line with SOCODEVI's guidelines on gender inclusion. Table 1 outlines the breakdown of surveyed members by farmers' associations and gender.

**TABLE 1:** Surveyed members by farmer associations and gender.

District	Farmers association name	Total members	Surveyed members		Total surveyed
			Women	Men	
Chókwè	Guinguiricane	32	8	2	10
	Armalugue	52	11	5	16
	Josina Machel	90	21	6	27
Mandlakazi	Esperança da Vida	14	5	2	7
	Árvore da Vida	23	9	2	11
	Lhuvukane	32	9	4	13
	Filipe Nyusi	22	7	2	9
Guijá	Nkateco	53	12	5	12
	Makateco	31	9	3	17
**Total**		**349**	**91**	**31**	**122**

### 3.3 Data Collection

Primary data collection took place between June and July 2024 through structured interviews conducted by trained enumerators who were fluent in both Portuguese (Mozambique's official language) and Changana (the local language spoken in the three districts). The questionnaire focused on several key areas: water access and availability, household and farm characteristics, institutional support and farmers' association networks, and coping strategies for water scarcity. Prior to the data collection, a pretest was conducted to refine the survey questions. Field observations were also carried out to assess irrigation infrastructure and water management practices. To foster collaboration and ensure the relevance of data, the research team engaged with district agricultural officers and extension services. This helped ensure that the data collection process aligned with the local context.

### 3.4 Statistical Analysis

The data collected were first entered into *Kobo Toolbox* and subsequently exported to Stata version 14.2 for analysis. Prior to analysis, the dataset was screened for completeness and consistency. Since all variables were categorical, the results are presented as frequencies and percentages. Cross‐tabulations were used to summarize and compare distributions across selected characteristics of the farmers. Given the descriptive focus of this study, no inferential statistical tests (e.g., t‐tests, chi‐square) were applied. Ethical considerations were strictly adhered to throughout the study. During data collection, environmental and social safeguards were observed, with an emphasis on preventing sexual harassment and abuse, as well as promoting inclusion through the random selection of interviewees. All participants provided informed consent, and the confidentiality of the data was maintained to ensure the privacy of the individuals involved.

## 4 Results and Discussion

Table 2 provides valuable insights into socio‐economic characteristics, water access, and irrigation challenges faced by smallholder farmers in the Chókwè, Mandlakazi, and Guijá districts. The demographic distribution reveals that most farmers are aged 45 years or older, with a significant proportion being over 60 years. This ageing farmer population suggests potential long‐term concerns regarding labor availability and sustainability in smallholder farming. Additionally, the data shows that women constitute the majority of respondents, reinforcing the essential role of women in smallholder agricultural activities. This finding aligns with studies that highlight the prominence of women in African agriculture and the need for gender‐inclusive policies to support their access to irrigation and other agricultural resources [29]. Water access remains a critical issue, with significant disparities between the three districts. Famers in Guijá are exclusively relying on river water, while those in Mandlakazi depend entirely on spring water. In contrast, farmers in Chókwè have access to a more diverse range of water sources, including rivers, lagoons, and public irrigation systems. This variation highlights differences in water infrastructure and resource availability across districts, reflecting broader challenges in water governance and distribution in Mozambique [30]. Despite these disparities, most farmers (82.79%) reported having sufficient water for irrigation; however, localized shortages and seasonal variations likely affect its reliability.

**TABLE 2:** Socio‐economic characteristics, water access, and irrigation challenges among smallholder farmers.

Variable	Chókwè (n = 53)	Mandlakazi (n = 40)	Guijá (n = 29)	Total (N = 122)
**Age group (%)**	—	—	—	—
18‐35 years	0.00	20.00	13.79	9.84
35‐45 years	16.98	12.50	20.69	16.39
45‐55 years	20.75	40.00	20.69	27.05
55‐60 years	26.42	15.00	20.69	21.31
Above 60 years	35.85	12.50	24.14	25.41
**Gender (%)**	—	—	—	—
Male	24.53	22.50	27.59	24.59
Female	75.47	77.50	72.41	75.41
**Main occupation (%)**	—	—	—	—
Farmer	100.00	100.00	100.00	100.00
**Primary water source (%)**	—	—	—	—
River	30.19	0.00	100.00	36.89
Lagoon	18.87	0.00	0.00	8.20
Spring	0.00	100.00	0.00	32.79
Public irrigation system	50.94	0.00	0.00	22.13
**Sufficiency of water supply (%)**	—	—	—	—
Yes	77.36	77.50	100.00	82.79
No	22.64	22.50	0.00	17.21
**Challenges during drought (%)**	—	—	—	—
Crop losses	26.42	12.50	0.00	15.57
Reduced yield	73.59	87.50	100.00	84.42
**Current irrigation method (%)**	—	—	—	—
Gravity flow	100.00	25.00	100.00	75.41
Buckets/watering cans	0.00	30.00	0.00	9.84
Sub irrigation/flooding	0.00	45.00	0.00	14.75
**Drainage system available (%)**	—	—	—	—
Yes	60.38	42.50	0.00	40.16
No	39.62	57.50	100.00	59.84
**Need for improvement**	—	—	—	—
Yes	100.00	82.50	96.55	93.39
No	0.00	17.50	3.45	6.61

The table also sheds light on the predominant irrigation methods used by smallholder farmers. Gravity flow irrigation is the most widely adopted system. However, Mandlakazi shows a higher reliance on less efficient methods such as sub‐irrigation and bucket or watering can irrigation, which may indicate inadequate access to proper irrigation infrastructure. Additionally, the predominance of sandy soils and the nature of the water sources, condition Mandlakazi to the irrigation methods previously listed. The dominance of gravity flow irrigation aligns with findings from Kadigi et al. [31], who noted that many smallholder farmers in sub‐Saharan Africa still rely on traditional irrigation systems, which often have low water‐use efficiency. The limited adoption of advanced techniques, such as drip irrigation, underscores the need for investment in water‐saving technologies to enhance irrigation efficiency and improve climate resilience [32]. Drought‐related challenges further exacerbate the vulnerabilities faced by smallholder farmers in the Gaza province. A significant number of farmers reported reduced yields due to drought, while some experienced complete crop losses. These challenges underscore the growing impact of climate variability on irrigation‐dependent farming, underscoring the need for enhanced water management strategies and effective drought adaptation measures. The high reliance on surface water sources, coupled with the lack of adequate storage facilities, makes smallholder farmers highly susceptible to seasonal water shortages, as noted by Payen et al. [33].

The most critical finding is that nearly all respondents expressed the need for improvement in irrigation infrastructure. This overwhelming demand for better irrigation systems suggests a pressing need for policy interventions that focus on strengthening water governance, securing land tenure, and providing financial support for irrigation investments [34]. Strengthening water user associations, improving access to rural infrastructure, and promoting climate‐smart agricultural practices could significantly enhance the resilience and productivity of smallholder irrigation farming in Gaza province [35].

Table 3 presents a breakdown of adaptation strategies adopted by smallholder farmers in the Chókwè, Mandlakazi, and Guijá districts to improve irrigation, water management, and climate adaptation. The data highlights key areas of intervention, including infrastructure improvements, equipment investments, water management strategies, climate adaptation practices, and capacity‐building initiatives. The variation observed across districts provides insights into the specific challenges and priorities faced by farmers in each region. Infrastructure improvements are a critical area of focus, particularly in Chókwè and Mandlakazi. Many farmers in Chókwè reported a need to rehabilitate gutters, suggesting that irrigation infrastructure in the district may be deteriorating and require urgent rehabilitation to improve water distribution efficiency. This finding is consistent with studies emphasizing the need for periodic maintenance of smallholder irrigation systems to ensure sustainable water use productivity [36]. Meanwhile, the construction of the main irrigation channel is a higher priority in Mandlakazi and Guijá than in Chókwè, indicating that these districts may lack adequate irrigation infrastructure. The expansion of irrigation channels is a key strategy for improving smallholder water access in sub‐Saharan Africa [37]. Similarly, farmers in Mandlakazi consider the construction of a main drainage system necessary, highlighting challenges related to waterlogging and inefficient drainage, which are commonly reported constraints in smallholder irrigation schemes [38].

**TABLE 3:** Farmer adaptation strategies for irrigation and climate resilience.

Variable	Chókwè (n = 53)	Mandlakazi (n = 40)	Guijá (n = 29)	Total (N = 122)
**Infrastructure improvements (%)**	—	—	—	—
Rehabilitate gutters	67.92	5.71	17.86	37.07
Construct main irrigation channel	32.07	80.00	49.99	37.07
Construct main drainage system	0.00	37.14	0.00	9.49
Rehabititate channels and drainage ditches	5.66	37.14	10.71	16.38
**Equipment improvements (%)**	—	—	—	—
Borehole with solar panel and piping	13.51	38.46	0.00	17.86
Motor pump and irrigation pipes	29.73	30.77	38.10	32.14
Motor pump	24.32	11.54	33.33	22.62
Irrigation pipes	2.70	3.85	23.81	8.33
**Water management and drainage solutions (%)**	—	—	—	—
Construct drainage ditch	42.38	47.00	95.00	54.83
Clean drainage ditch	9.52	0.00	5.00	5.21
Rehabilitate drainage and ditches	42.86	38.24	0.00	32.29
Rehabilitate drains	4.76	14.71	0.00	7.29
**Climate adaptation and agricultural practices (%)**	—	—	—	—
Conservation agriculture	47.62	30.77	5.88	33.67
Use of drought‐tolerant crops	35.71	25.64	76.47	38.78
Reduction in the number of crops	16.67	43.59	17.65	27.55
**Capacity building and knowledge development (%)**		—	—	—
Principles of sustainable water management	2.22	8.11	0.00	3.85
Managing water scarcity and natural disasters	15.56	91.89	18.18	43.27
Training in irrigation scheduling	82.22	0.00	81.82	52.88

Regarding equipment improvements, farmers across all three districts recognize the importance of investing in irrigation technology. The need for boreholes with solar panels and piping is particularly high in Mandlakazi, suggesting challenges in accessing a reliable water source. This finding supports previous studies that emphasize solar‐powered irrigation as a sustainable solution for smallholder farmers facing water access constraints [39]. The demand for motor pumps and irrigation pipes is widespread, with Guijá showing the highest need for irrigation pipes alone, possibly indicating that some farmers already own motor pumps but lack proper distribution systems. Research indicates that improving access to irrigation equipment, particularly solar‐powered pumps and efficient piping systems, can enhance water use efficiency and climate resilience in water‐scarce regions [40]. Water management and drainage solutions remain a crucial aspect of mitigation strategies. Farmers in Guijá overwhelmingly highlight the need for constructing new drainage ditches, likely due to severe water retention issues. Studies have shown that inadequate drainage contributes to soil degradation and reduced crop yields, making improved drainage systems a key adaptation strategy for climate‐vulnerable smallholder farmers [41]. Meanwhile, the rehabilitation of existing drainage ditches is a priority in both Chókwè and Mandlakazi districts, reinforcing the need for improved water flow management. Previous research confirms that well‐maintained drainage infrastructure can significantly reduce the risk of waterlogging and improve soil fertility in irrigation‐dependent farming systems [42].

Climate adaptation and agricultural practices play a vital role in ensuring resilience to climate variability. Conservation agriculture is more commonly practiced in Chókwè and Mandlakazi, while fewer farmers in Guijá report using this technique, indicating a need for greater support in adopting sustainable soil and water conservation methods. This finding is consistent with studies showing that conservation agriculture enhances soil moisture retention and reduces vulnerability to erratic rainfall [43]. Conversely, the use of drought‐tolerant crops is a high priority in Guijá, reflecting more severe drought conditions in the district. Research has demonstrated that integrating drought‐tolerant crops into smallholder farming systems can improve food security and reduce vulnerability to climate change [44]. In Mandlakazi, reducing the number of cultivated crops is a common strategy, suggesting it may be a coping mechanism against limited water availability, a strategy widely documented in drought‐prone agricultural systems [45]. Capacity building and knowledge development are essential for improving farmers' ability to manage water resources effectively. Training on managing water scarcity and natural disasters is particularly needed in Mandlakazi, highlighting the district's exposure to extreme weather events. This aligns with broader findings emphasizing the importance of disaster preparedness training for climate resilience in smallholder farming [46]. In contrast, farmers in Chókwè and Guijá show a stronger demand for training in irrigation scheduling, which could help improve water use efficiency in these regions. Studies have demonstrated that improved irrigation scheduling can optimize water use, reduce waste, and enhance agricultural productivity in water‐scarce environments [47].

## 5 Conclusion and Implications

This study presents a comprehensive analysis of the irrigation challenges, water access issues, and adaptation strategies employed by smallholder farmers in Gaza province, Mozambique. The findings highlight several critical issues that hinder adequate irrigation, including ageing farmers, gender disparities, inadequate infrastructure, and climate variability, all of which are exacerbated by a lack of sufficient water storage and drainage systems. Despite these challenges, farmers have implemented various adaptation strategies, including improving water management, investing in irrigation technologies, and adopting climate‐resilient practices such as drought‐tolerant crops and conservation agriculture. The need for infrastructure improvement is particularly urgent, with nearly all respondents indicating the need for better irrigation systems. The study found that smallholder farmers in the region rely heavily on gravity flow irrigation, a practice that reflects and reinforces the broader challenge of inadequate irrigation infrastructure. The absence of reliable water storage, modern pumping technologies, and effective drainage channels compels farmers to depend on gravity‐fed systems, which are often inefficient and highly vulnerable to seasonal variability. This reliance not only limits water availability but also constrains the adoption of more efficient technologies, such as solar‐powered pumps and advanced piping systems, and delays optimal water use. Furthermore, the prevalence of drought‐related crop losses highlights the importance of adopting water‐saving technologies and improving water management practices. Farmers in the study districts also emphasized the need for capacity building, particularly in areas such as irrigation scheduling and disaster preparedness, to strengthen their resilience to climate‐related challenges.

The findings of this study suggest several key policy recommendations to address the irrigation challenges and improve water access for smallholder farmers in Gaza province. First, there is a critical need for investment in irrigation infrastructures across Gaza province, particularly in the rehabilitation of existing systems, the construction of new irrigation channels, and the establishment of effective drainage systems. This would enhance water distribution and reduce the risks of waterlogging and inefficiencies in irrigation practices. Second, policymakers should prioritize facilitating access to water‐saving technologies such as solar‐powered pumps and efficient irrigation piping systems. These technologies could significantly improve water use efficiency and reduce dependence on unreliable water sources, thus increasing the resilience of smallholder farming to climate change. Third, improved governance and better water resources management are essential to ensure equitable distribution of water. Strengthening water user associations and enhancing local governance mechanisms can help improve access to water, especially in regions with significant disparities in water sources. Fourth, training programs focused on sustainable water management, irrigation scheduling, and climate adaptation practices should be expanded to ensure that farmers have the necessary knowledge and skills to cope with water scarcity and extreme weather events. This would increase the efficiency of water use and improve the overall productivity of smallholder farmers. Lastly, the study highlights the significant role of women in smallholder farming in Gaza province. Policies must be designed to be gender‐inclusive, ensuring women have equal access to irrigation resources, technology, and decision‐making processes. Supporting women's participation in water management could further enhance agricultural productivity and food security.

While the findings offer valuable insights into the challenges and adaptation strategies of smallholder farmers in Gaza province, the study has some limitations. First, the research does not represent the entire population of Gaza province, as it was conducted in only three districts. Consequently, the experiences and challenges of smallholder farmers in other areas of the province may not be fully captured. Second, farmers' associations were identified with the support of SOCODEVI, an international organization working on the Mozambique Rural Women's Economic Empowerment project. The selection of participants within these associations may introduce some bias, as it focuses on farmers who are part of these organizations, potentially overlooking those who are not engaged in such groups. Third, the study is based on cross‐sectional field data, which captures farmers’ adaptation strategies at a single point in time. As a result, it does not reflect how these strategies evolve in response to long‐term climate variability, infrastructural changes, or shifts in institutional support. Future research should therefore adopt longitudinal or historical approaches to capture temporal trends in adaptation dynamics more effectively and provide a more robust understanding of how farmers’ strategies change over time. Expanding both geographic scope and temporal depth of analysis would offer a more comprehensive picture of irrigation challenges and adaptive responses in Gaza province and beyond (Supporting Information).

## Funding

The Society funded this study for the Society for Cooperation in International Development (SOCODEVI) under Contract 1/2024 and Addenda Nos. 1, 2, and 3 of the Professional Services Agreement.

## Conflicts of Interest

The authors declare no conflict of interest.

## Supporting information




**Supporting Information file**: gch270074‐sup‐0001‐SuppMat.xlsx

## Data Availability

The data that support the findings of this study are available from the corresponding author upon reasonable request.
